# Describing the whiskers of a semi-aquatic caniform: the Eurasian Otter (*Lutra lutra*)

**DOI:** 10.1093/jmammal/gyag021

**Published:** 2026-04-09

**Authors:** Amisha A Nakhwa, Vicki Breakell, Lewis Chambers, Sally Holt, Sarah Roberts, Robyn A Grant

**Affiliations:** Department of Natural Science, Manchester Metropolitan University, Manchester M1 5GD, United Kingdom; Oceans and Coasts Programme, Nature Conservation Foundation, Mysore 570017, India; The Wildwood Trust, Herne Common, Kent CT6 7LQ, United Kingdom; The Wildwood Trust, Herne Common, Kent CT6 7LQ, United Kingdom; The Wildwood Trust, Herne Common, Kent CT6 7LQ, United Kingdom; The Wildwood Trust, Herne Common, Kent CT6 7LQ, United Kingdom; Department of Natural Science, Manchester Metropolitan University, Manchester M1 5GD, United Kingdom

**Keywords:** Carnivora, Lutrinae, somatosensory, touch sensing, vibrissae

## Abstract

Recent research has identified specializations in whisker shape and material in aquatic species, such as pinnipeds, that serve to increase the stiffness of their whiskers to allow for precise positioning underwater. The whiskers of semi-aquatic species are thought to be somewhat intermediary between aquatic and terrestrial species, but have been fairly overlooked, especially when compared to pinnipeds, which are easily trained and widely available in zoological collections. In this study, we describe the whiskers of the Eurasian Otter (*Lutra lutra*), including their shape, layout, musculature, movement, and control behaviours. *Lutra lutra* have 38 to 43 long mystacial whiskers. Taper, base width, and length are strong predictors of whisker shape. Whisker arrangement varies somewhat between individuals, especially in the 5 dorsal-most rows, which are rather disorganized with regard to both layout and intrinsic musculature. The whiskers can be protracted underwater using intrinsic muscles, as well as adapted extrinsic muscles (Pars interna profunda and Pars maxillaris). Whisker movements are common in *L. lutra*, as well as other contact-related control behaviors, including contact-induced asymmetry. The large numbers of whiskers, the regularity of whisker movements and specializations in whisker shape and muscles demonstrate the importance of whisker sensing in *L. lutra*. Despite this, *L. lutra* whiskers lack any adaptations in cross-sectional shape, such as oval shapes and undulations that occur in phocids. Nevertheless, studying the whiskers of semi-aquatic mammals reveals their specialized sensory adaptations to the aquatic environment, and can give us insights into the evolution of mammalian somatosensory systems.

## Teaser

Studying the whiskers of the semi-aquatic Eurasian Otter (*Lutra lutra*) has revealed interesting adaptations similar to what we have previously observed in pinnipeds, including thick, strongly tapered whiskers to make whiskers stiffer and easier to position underwater, as well as large extrinsic muscles to drive whisker protractions. However, *L. lutra* whiskers lack any adaptations in cross-sectional shape, such as the oval shapes and undulations that we see in phocids. Nevertheless, the large numbers of whiskers, the regularity of whisker movements and specializations in whisker shape and muscles demonstrate the importance of whisker sensing in *L. lutra*.

**Fig. 1:**
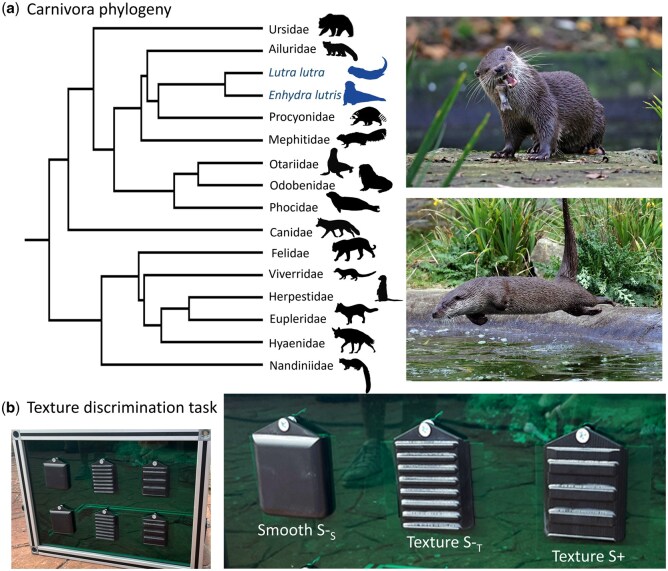
Carnivora phylogeny and texture discrimination task. a) Often pinnipeds are studied for whisker sensing (including both Otariidae and Phocidae), and the otters (Mustelidae: Lutrinae, in blue) are often overlooked, with only the Sea Otter (*Enhydra lutris*) and Eurasian Otter (*Lutra* lutra) previously studied a little. This is despite their whiskers showing a diversity of positions during feeding (top right) and locomoting (bottom right). b) The texture discrimination task, and a close-up of the stimuli from the task, including a textured S+ stimulus, and 2 S– stimuli (smooth and textured).

The order Carnivora includes the suborder Caniformia—a diverse group of terrestrial, aquatic, and semi-aquatic mammals, including dogs, bears, raccoons, mustelids, and pinnipeds (Fig. 1a). All caniforms possess facial vibrissae, or whiskers, which are specialized vibrotactile sensors that contribute to guiding key behaviors such as navigation, locomotion, foraging, and social interactions (Grant and Arkley 2016; Dougill et al. 2020; Grant and Goss 2022; Dougill et al. 2023). The evolution of aquatic lifestyles in caniforms has driven morphological adaptations in the aquatic pinnipeds (seals, sea lions, and walruses; Dehnhardt and Hanke 2018), the semi-aquatic mustelids (otters and minks; Botton-Divet et al. 2017), and ursids (polar bears; Slater et al. 2010), particularly in whisker structure and function (Dougill et al. 2020, 2023). Comparative studies have found aquatic mammals to have more sensitive whiskers than their terrestrial counterparts (Dykes 1975; Hyvärinen et al. 2009), reflecting a greater reliance on tactile sensing in species that inhabit dark, complex underwater environments (Grant and Arkley 2016).

In mammals, whisker shape is grossly similar—being long, slender, tapered hairs (Williams and Kramer 2010; Hires et al. 2013; Belli et al. 2017) with an inherent curvature in one plane (Starostin et al. 2020; Luo and Hartmann 2023) and a circular cross-section. However, these geometric properties can vary somewhat between species. Specifically, aquatic caniforms such as pinnipeds generally possess shorter, thicker whiskers compared to their terrestrial relatives, which makes their whiskers stiffer to allow precise positioning underwater (Dougill et al. 2020, 2023; Grant et al. 2025). Pinniped whiskers can also be oval in cross-section and undulating along their length, which is thought to be important for efficient hydrodynamic sensing (Hanke et al. 2010; Ginter et al. 2012; Milne et al. 2022). Pinniped whiskers are commonly studied, owing not only to their highly specialized whisker shape but also their ease of being accessed and trained in captive settings (Elder et al. 2025b). This has led to semi-aquatic species such as the Eurasian Otter (*Lutra lutra*) being scarcely studied, despite them also being trainable and found in many captive collections. Indeed, so far, whisker studies on *L. lutra* have been limited to being incorporated into broad comparative analyses rather than included in any species-specific investigations.

Recent findings indicate that semi-aquatic species such as *L. lutra* and American Mink (*Neogale vision*) possess intermediate values of whisker length, thickness, and stiffness, falling between the values of aquatic and terrestrial caniforms (Dougill et al. 2020, 2023; Grant et al. 2025), although this pattern has not yet been investigated in depth. The semi-aquatic Lutrinae subfamily (Fig. 1a) includes 13 extant species of otters across 7 genera that exhibit diverse foraging strategies ranging from piscivorous, mouth-oriented predation to hand-oriented invertebrate predation in aquatic environments (Radinsky 1968; Sivasothi and Nor 1994; Jacques et al. 2009). Among them, the Sea Otter (*Enhydra lutris*), which is most specialized for a marine lifestyle, is known to rely heavily on tactile sensing using its forelimb paws and facial whiskers to locate and capture cryptic prey underwater (Strobel et al. 2018). *Lutra lutra* whisker movements have been measured during novel object (Grant et al. 2023) and texture discrimination tasks (Nakhwa et al. 2024), with whisker movements being common (see Fig. 1a, right panels) but smaller than those observed in pinnipeds (Nakhwa et al. 2024). However, there are many types of whisker behaviors beyond simple protraction (forward) movements that enhance tactile perception by allowing animals to modulate whisker positioning based on environmental contacts, including contact-induced asymmetry (CIA; Mitchinson et al. 2007) and spread reduction (Grant et al. 2009)—both thought to increase the number of whisker contacts during surface exploration (Grant and Arkley 2016)—but these behaviors have not yet been described in otters.

Facial musculature plays a crucial role in controlling whisker behaviors. The gross whisker muscle architecture has been conserved in mammals, from marsupials to primates (Grant et al. 2013b; Muchlinski et al. 2013), and includes both intrinsic and extrinsic muscles. Intrinsic muscles form a sling around every whisker follicle and control protraction (Dörfl 1982; Haidarliu et al. 2024). Extrinsic muscles attach outside of the pad and translate the whole pad, controlling aspects of protraction, retraction, and spread (Haidarliu et al. 2024). Due to the added drag of protracting whiskers underwater, we might expect interesting adaptations in the intrinsic or extrinsic muscles responsible for whisker protraction in *L. lutra* that have not been investigated before. Indeed, of all aquatic and semi-aquatic mammals, only the whisker muscles of harbor seals (*Phoca vitulina*) have been described so far (Elder et al. 2025a). Further investigation into the detailed structure and function of whiskers of a semi-aquatic species would provide deeper insights into their sensory adaptations.

Despite extensive research on the whisker systems of pinnipeds and sea otters, *L. lutra* remains relatively understudied, particularly in terms of its whisker movements, sensory control, and the underlying musculature involved in whisker kinematics. In this study, we will provide the first description of the whisker shape, layout, and musculature within the mystacial pads of *L. lutra*. We investigate whisker behaviors and control, and will discuss the musculature responsible for modulating whisker movements during tactile exploration. Understanding these aspects will contribute to a more comprehensive overview of sensory adaptations in semi-aquatic mammals and refine our knowledge of whisker structure and function.

## Methods

### Whisker morphology

Five individual specimens of *L. lutra* (museum IDs: GH40.22, GH80.22, 3× unregistered) were loaned by National Museums Scotland. All specimens were stored frozen at the museum, and once defrosted, the mystacial pads were dissected from the specimens and stored in 10% formalin until further processing. After a few weeks in formalin, the most intact whisker pad was selected from each specimen. Anecdotally, we have not observed any changes in whisker shape with formalin compared to freezing. The selected pad was plucked, and each whisker was labelled in terms of whisker row (with the most dorsal whisker being labelled row A) and column (with the most caudal whisker being labelled column 1). Some whiskers showed signs of damage, including split or cut ends that likely occurred in a living animal, but also included whiskers that were bent during plucking. Any whiskers with signs of damage were rejected from the study and were judged by investigating the physical specimens and scans, especially the whisker shaft to ensure a smooth shape (without any kinks); and the whisker tip to ensure a curved ending, with no abrupt blunt end, or splitting. This procedure resulted in the removal of 11 whiskers from the analysis.

Two hundred and seven whiskers were scanned from 5 adult individuals of *L. lutra*, ranging from 38 to 43 whiskers per individual. There has been no evidence for age or sex differences in whisker morphology studies (Ginter Summarell et al. 2015; Grant et al. 2018), so whiskers were assumed to be sexually monomorphic. Scanning was undertaken using an Epson V600 photo-scanner (Epson, Tokyo, Japan) to gather 2-dimensional whisker shape parameters of curvature, length, width, and taper. Image resolutions were between 2 and 8 µm. Before scanning, each whisker was rolled between a thumb and finger to assess the roundness of its cross-sectional shape. Whiskers were then laid flat on the scanner bed in their unstressed state, and shapes were extracted from the outlines of the scanned images and processed as previously described in Starostin et al. (2020) and Dougill et al. (2020; 2023) using 100 key points. A full explanation of this process can be found in the Supplementary Material SM2 in Dougill et al. (2020). Following Dougill et al. (2020), we extracted 2 measures of whisker curvature: A and B, absolute whisker length (mm), width (base radius normalized to whisker length), and taper gradient (coefficient ω1). A principal component (PC) analysis was conducted on the standardized data. Two example whiskers were also cut, just above the bulb, and imaged on a light microscope to further evaluate their circular cross-sectional shape.

### Whisker layout and musculature

One of the 5 remaining unplucked mystacial pads was embedded in paraffin and sectioned into 10 µm sections, then stained with Masson’s Trichrome, which turns muscles red and collagen blue. Follicle arrangements from the histology were also checked against the remaining 4 plucked and labelled whisker specimens. An additional unplucked mystacial pad was Dice-CT scanned (as per the protocol of Evans and Elder 2024) to confirm the muscle positions from histology.

### Whisker behavior

A 7-year-old male *L. lutra* named Loki, housed at the Wildwood Trust, United Kingdom was used for the behavioral study. Loki had prior experience with enrichment tasks and regular target training. A tactile, texture discrimination enrichment device (Nakhwa et al. 2024) was used to encourage whisker movements. The device consisted of a metal frame with an acrylic sheet containing 6 square slots, each fitted with a movable door. These doors presented different tactile textures: 2 smooth (no grooves); 2 with fine grooves (5 mm apart, 2 mm deep); and 2 with wider grooves (10 mm apart, 2 mm deep). The 10 mm grooved doors served as target stimuli (S+), while the others functioned as distractors (S-_T_ as a textured distractor, S-_S_ as a smooth distractor; Fig. 1b). The position of the doors was decided quasi-randomly before each trial to ensure no side or height preferences. Food rewards (e.g., pieces of chick, rat, ox heart, or fish) were placed behind the target-textured doors (S+; Fig. 1b) to reinforce the choice of the target texture.

Data collection began for this study after 4 wk, once the otter had learned the texture task. A GoPro camera (120 fps) was mounted above the apparatus to capture whisker movements from a top-down perspective, a standard method for tracking whisker movements (Grant et al. 2023). A trial was defined when the otter approached and made whisker contact with 1 of the stimuli (S+ or S–). A total of 23 trials were analyzed. Whisker control behaviors were assessed from the footage. The behaviors were scored based on scales developed in Grant et al. (2018). Whisker movements were scored either as absent (0), only retractions present (1), or retractions and protractions present (2); spread reduction as absent (0) or present (1); and contact-induced asymmetry as absent (0), only an ipsilateral decrease (1), or an only an ipsilateral decrease and contralateral increase (2).

In each trial, the nose tip—a point between the eyes and 3 whiskers on both sides of the face (in 5 trials)—was tracked using the “Manual Whisker Annotator” (MWA) software (Hewitt et al. 2016). In 18 trials, only 1 side of the face was visible, so for these trials, only 1 side of the face was tracked and combined with the bilateral whisker tracking data. For each whisker, 2 points were tracked: the base and a point two-thirds along its length. The following whisker metrics were calculated: mean angular position (mean angle of all tracked whiskers across frames); maximum amplitude (difference between the maximum and minimum whisker angles from the mean angular position); and whisker asymmetry (difference between the mean angles of the left and right whiskers, when data for both sides were available; Grant et al. 2023). Manual tracking is often considered the “gold standard” of tracking (Gillespie et al. 2019; Gill et al. 2025), and our manual tracking had low error rates of ∼2 pixels, equating to <1 degree error (Grant et al. 2009). Decision time was also recorded for each trial, from the first whisker touch with the door to the point of either choosing the door by moving it or moving away from the door.

To test whether whisker movement variables (mean whisker offset, maximum amplitude, and mean asymmetry) and decision times differed across stimulus types, we used permutation-based linear models. Due to the small sample size (*n *= 23 clips and *n *= 1 individual) and non-normality of data, standard comparative ANOVA tests following parametric assumptions could not be used. A model was constructed with texture stimulus types S-_T_ (*n *= 7), S-_S_ (*n *= 7), and S+ (*n *= 9) as the fixed factors. Post hoc pairwise permutation comparisons were conducted when significance was observed between more than 1 pair of dependent variables. To control for multiple testing, we applied a Bonferroni correction, giving a significance level of 0.017.

All statistical analyses were conducted using base R and the package lmerPerm (R version 4.3.3; R Core Team, 2024). All procedures were approved by Manchester Metropolitan University Faculty of Science and Engineering ethical committee (ID 6009) and the Wildwood Trust.

## Results

### Whisker shape

**Fig. 2:**
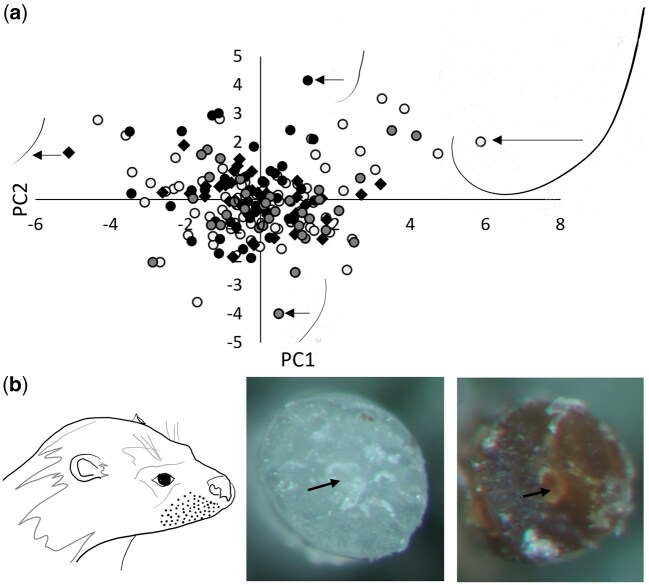
Principal component (PC) analysis of Eurasian Otter (*Lutra lutra*) using whisker shape metrics (curvature, base radius, taper gradient, and length); a) scatterplot of PC1 (50%) and PC2 (33%) values. Each individual specimen is coded as a different shape or color. Example whiskers show “extreme” examples of PC1 and PC2. b) Cross-section through 2 example whiskers; the arrow indicates the medulla.

When a PCA was conducted on the whisker shape metrics curvature (A and B), base radius, taper gradient (ω1), and length (Table 1), the first 2 PCs accounted for 83% of the variance (50% and 33%, respectively; Table 1; Fig. 2a). There was a large reduction in variance values between PC2 (33%) and PC3 (9%), suggesting that the first 2 components were sufficient for capturing the variation in the data. There was a lot of overlap of PC1 and PC2 values between individuals (Fig. 1a; Supplementary Data SD1). PC1 was explained mainly by whisker base radius, length, and taper (loadings ∼0.5; Table 1), where the whisker representing the minimum value of PC1 was short and thin and the whisker representing the maximum value of PC1 was long and thick (Fig. 2a). PC 2 was explained mainly by the 2 curvature values (loadings ∼0.6; Table 1), where the whisker representing the minimum value of PC2 curved more at the base (top right of image) and less at the tip (bottom left of image), whereas the whisker representing the maximum values of PC2 curved more at the tip and less at the base (Fig. 2a).

**Table 1:** Principal component analysis of whisker shape metrics showing the variance explained by each of the principal components (PC1-5), as well as the loadings of each metric.

	Mean ± SD	PC1	PC2	PC3	PC4	PC5
**Variance**		**0.50**	**0.33**	0.086	0.051	0.032
**Curvature A**	0.25 ± 1.10	0.37	0.57*			
**Curvature B**	0.23 ± 0.50	–0.31	–0.62*			
**Base radius**	0.01 ± 0.00	–0.51*	0.36			
**Taper ω1**	–0.01 ± 0.00	0.48*	–0.40			
**Length**	23.50 ± 14.00	0.52*	–0.06			

Bold indicates contributing metrics to the PC (0.5 and above). Mean and SDs for each input metric can also be seen in column 2. Breakdowns of each variable for individual otters (with higher decimal places) can be seen in Supplement Data SD1.

When each of the whiskers was rolled between the thumb and finger, it was clear that they all had circular cross-sectional shapes, lacking the oval shape found in pinniped whiskers. To further demonstrate this, in 2 whisker samples that were cut around 3 mm above the bulb of the whisker at the base, the cross-section was circular in both examples (Fig. 2b). The whiskers both contained a clear medulla region (arrow in Fig. 2b) and outer cuticle and cortex.

### Layout and musculature

**Fig. 3:**
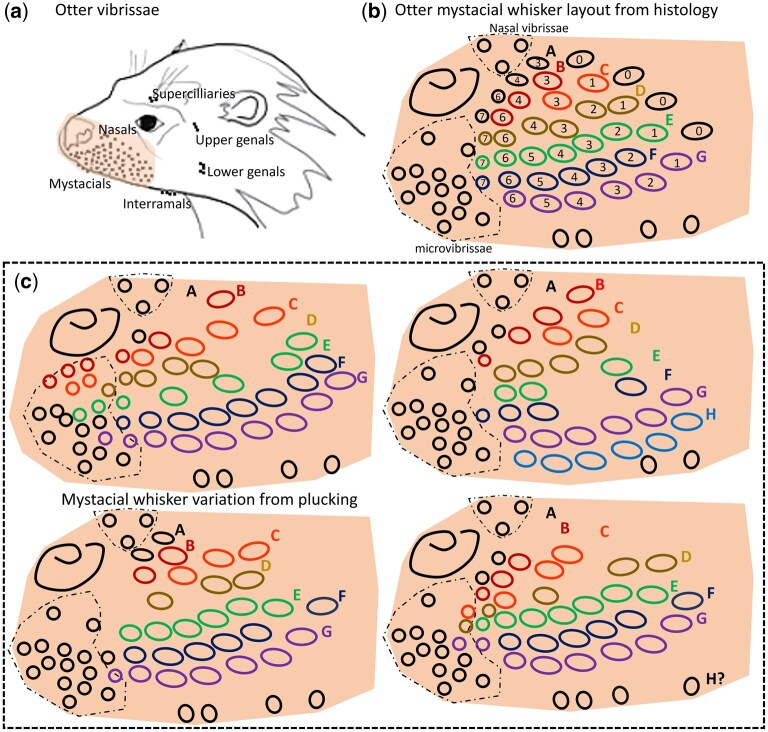
Layout of whiskers in 5 Eurasian otters (*Lutra lutra*). a) Vibrissal groupings observed in the specimens; b) an example mystacial whisker layout from observing the histology, including the macrovibrissae (numbered), microvibrissae, nasal vibrissae, and follicles ventral to the pad following the top lip; c) mystacial whisker layouts from plucking show some variation between them.

Dissection of the mystacial pads showed that *L. lutra* had many other vibrissal groups in addition to the mystacial whiskers including the supercilliaries (above the eye), upper and lower genals (caudal to the mystacial pad) and interammals (below the chin) (Fig. 3a). The genal whiskers were especially long, with some longer than the mystacial whiskers. Within the mystacial pad, some whiskers sat outside of the main muscular pad, including nasal whiskers (dorsal to the mystacial pad), microvibrissae (rostral to the mystacial pad) and some other whiskers ventral to the pad, following the upper lip line (Fig. 3b). All these whiskers were small in length and tended to vary in number and position between individuals. It was challenging to determine whether they sat external to, or within, the mystacial pad muscles without doing further histology.

**Fig. 4:**
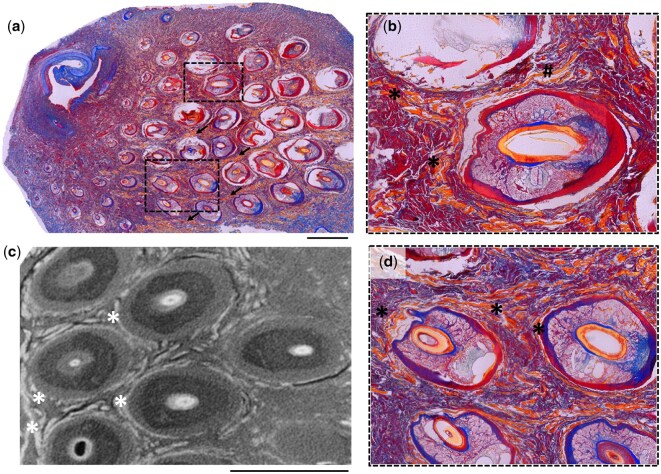
Intrinsic musculature of the mystacial pad of Eurasian Otter (*Lutra lutra*). a) A sagittal cross-section across the pad, showing individual follicles and some intrinsic muscles. Inset b) shows intrinsic muscles in the dorsal, more disorganized area of the pad, where the intrinsic muscles of 2 whiskers from different rows touch. Inset d) shows intrinsic muscles within the more ventral organized area, with intrinsic muscles of 2 neighboring follicles within the same row joining. Panel c shows a micro CT image of the follicles and intrinsic muscles from a dorsal area of the pad, also showing the touching of the intrinsic muscles between rows. Asterisks (*) indicate the intrinsic muscles; hashes (#) the irregular intrinsic muscles; and arrows point to the extrinsic Pars maxillaris muscles. Scale bars are 10 mm.

When we compared whisker arrangements between the plucked examples and the histology and CT scans, we did not observe differences between the left and right sides of the pad, suggesting that arrangements are roughly symmetric within the same individual. However, there was variation between the whisker arrangements and numbers between individuals (Fig. 3b and c). There were 7 whisker rows in all samples (note that row H in Fig. 3c is likely to sit external to the mystacial pad and should not be included in the mystical whisker count) and approximately 8 whisker columns (columns 0 to 7). An example mystacial pad with corresponding numbering generated during the histology process can be seen in Fig. 3b. The ventral 3 rows of the pad (E to G in Fig. 3b), were regularly arranged. The dorsal four rows (A to D in Fig. 3b and c) were often disorganized, and it was challenging to follow the whiskers along rows, which may have caused some of the observed variation in whisker rows and columns in Fig. 3c that occurred during plucking. For example, in Fig. 3b, we identified row C as having only 2 whiskers (1 and 3). Spatially, we may assume that B4 might also be part of row C, although looking at the underlying musculature (Fig. 4a) suggested that B4 was part of the B-row, and the C row was separate.

**Fig. 5:**
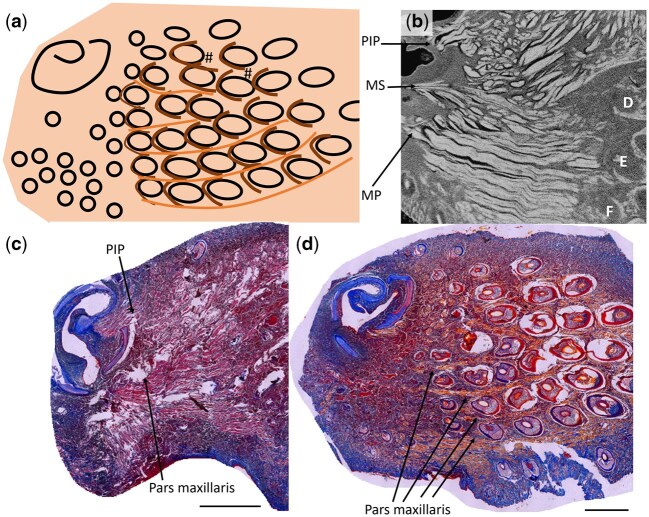
Extrinsic musculature of the mystacial pad of Eurasian Otter (*Lutra lutra*). a) An overview diagram of the muscles within the pad. b) A micro CT scan showing rostral and deep attachments of the Pars interna profunda (PIP), as well as the Pars maxillaris, including the Pars maxillaris superficialis (MS) and the Pars maxillaris profunda (MP); whisker follicles can also be seen, corresponding to rows D, E, and F and including columns 1 and 2. c) A similar view to panel b, showing the rostrally attached extrinsic muscles, including the Pars interna profunda, and the Pars maxillaris. The Pars maxillaris can also be seen in more superficial layers of the pad, between the whisker rows, especially the 3 most ventral rows (Panel d). Scale bars are 10 mm.

Both intrinsic and extrinsic muscles could be seen with the mystacial pad (Figs. 4 and 5). In the dorsal section of the pad (dorsal 4 rows in Fig. 4a), the arrangement of follicles was less organized, and consequently the intrinsic muscles were also less organized; sometimes even touching within different rows (Fig. 4b and c). In the ventral region of the pad (ventral 3 rows; Fig. 4a), the sling-like intrinsic muscles were organized and formed a regular sling around the most rostral follicle and connected it to the neighboring caudal follicle (Fig. 4a and d). Oblique intrinsic muscles were also observed in the more ventral rows, indicated by the asterisk (*) between the follicles in Fig. 4d. The caudal row of the pad contained straddler whiskers, which straddled 2 whisker rows and received muscle fibers from the neighboring rostral whiskers of both rows.

In the deeper layers of the pad, we observed extrinsic muscles that attached rostral to the pad, around the nose (Fig. 5a to c), including the more dorsal Pars interna profunda and the more ventral Pars maxillaris (Fig. 5b and c), which was made up of both the Pars maxillaris profunda and the Pars maxillaris superficialis (Fig. 5b). From the deep rostral layers, these muscles also appeared more superficially, running between the rows of the whisker follicles as well as ventral to the most ventral row (Fig. 5d) and were especially present along all the columns of the 3 most ventral rows, and in the rostral area of the more dorsal rows (Figs 4a and 5d). We did not observe any clear superficial extrinsic muscles towards the caudal area of the pad.

### Whisker behavior

**Fig. 6:**
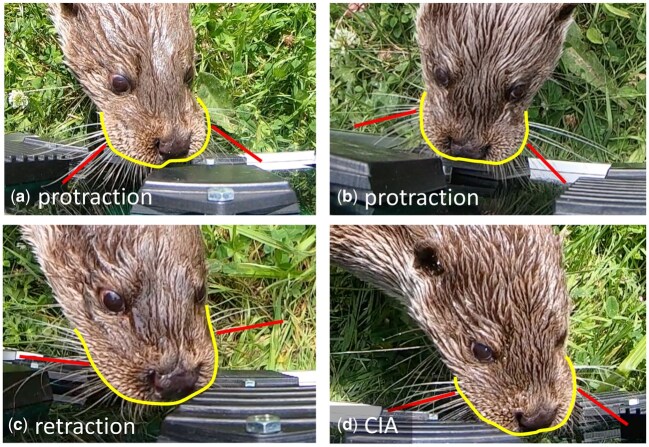
Whisker behavior examples. Example whisker control behaviors are shown here, including protraction (a and b), retraction (c), and contact-induced asymmetry (CIA; d). The yellow line shows the silhouette of the face, and the 2 red lines correspond to 2 middle whisker angular positions.

In most trials during the texture discrimination task, the otter approached with its head perpendicular to the stimulus, positioning its rostral whiskers to make contact (Fig. 6a and b). Controlled whisker movements such as protraction, retraction, or contact-induced asymmetry were observed to occur only after the initial whisker contact with the stimulus. Whisker movements were not always observed, but when they were present, they included a full range of protraction and retraction movements (44%; Fig. 6a, b, and c), with only retraction movements seen 4% of the time (Table 2). Spread reduction was not observed during any of the trials. When contact-induced asymmetry was observed (57%), it mainly involved a reduction in whisker angles on the ipsilateral side (39%), but occasionally included an increase in angles on the contralateral side (4%; Fig. 6d; Table 2).

**Table 2:** Whisker control behavior scores.

Score	Whisker movements (%)	Spread reduction (%)	Contact-induced asymmetry (%)
**0**	52.20	100.00	56.50
**1**	4.30	0.00	39.10
**2**	43.50	N/A	4.40

Whisker movements were scored either as absent (0), only retractions present (1), or retractions and protractions present (2); spread reduction as absent (0) or present (1); and contact-induced asymmetry as absent (0), only an ipsilateral decrease (1), or an only an ipsilateral decrease and contralateral increase (2).

Across all trials, the mean whisker angular position was 86.13 ± 4.78°, the mean maximum amplitude was 23.76 ± 14.34°, and the mean asymmetry was 12.82 ± 6.57°. The whisker movement variables (offset and amplitude) did not show any significant difference between the texture stimulus types (all *P *> .017; Table 3). However, decision times differed significantly between the different textures (*F*_2,17_ = 15.68, *P *< .001; Table 3). Pairwise permutation tests revealed that decisions were significantly faster for the smooth S-_S_ stimulus (0.098 ± 0.011 s) than for both textured S+ (0.131 ± 0.019 s; Table 3) and S-_T_ stimuli (0.132 ±0.038 s). Decision times did not differ significantly between S+ and S-_T_ doors (*P *> .017). Across all trials, the otter took less than 0.2 s to make a decision.

**Table 3:** Whisker movement and decision times during a texture discrimination task.

Stimuli	Mean angular position (°)	Amplitude (°)	Asymmetry (°)	Decision times (s)
**Textured S+**	84.0 ± 19.5	22.1 ± 8.0	22.88 ± 0.00	0.13 ± 0.02
**Textured S–**	87.9 ± 19.5	28.6 ± 21.6	12.53 ± 0.45	0.13 ± 0.04
**Smooth S–**	86.1 ± 14.9	20.4 ± 9.4	8.07 ± 5.10	0.098 ± 0.011
**Permutation linear model**	*F*(2,17) = 0.1 *P *= 0.91	*F*(2,17) = 0.62 *P *= 0.55;	N/A	*F*(2,17) = 15.69 *P *< 0.001
**Pairwise comparison**	N/A	N/A	N/A	S+, S-_T_ > S-_S_

S+ corresponds to the S+ textured stimulus, S-_T_ is the textured S– stimulus, and S-_S_ is the smooth S– stimulus.

## Discussion

By investigating the whiskers of *L. lutra*, we find that whisker base radius, length, and taper contribute to explaining most of the variance related to whisker shape, which are adaptations that we have previously identified as being associated with the aquatic environment. In our *L. lutra* specimens, we did not observe especially large intrinsic muscles to enable protractions underwater, but rather prominent extrinsic muscles (especially the Pars maxillaris) that drive the whiskers to protract underwater. We observed regular, large whisker movements during tactile discrimination as well as contact-induced asymmetry, a common whisker control behavior that has been documented in many species. Our observations that *L. lutra* have large whisker base radii, steep whisker taper gradients, and prominent extrinsic muscles and whisker movements are qualities that have only previously been observed in phocid whisker specialists. Our findings support the importance of studying the whiskers of Lutrinae. In particular, we suggest studying other species of Lutrinae to see if these qualities are present across the genus, as well as other pinnipeds, including otariids, to improve our understanding of aquatic adaptations in whisker morphology.

### Whisker musculature and control

Moving the whiskers underwater is energetically costly (Adachi et al. 2022), which is probably why aquatic and semi-aquatic species do not cyclically move their whiskers like many terrestrial mammals do (Grant and Arkley 2016; Muchlinski et al. 2020). However, we observed that *L. lutra* make clear protraction, retraction, and asymmetric whisker movements (Fig. 6). Whisker protractions are likely to be partly driven by the intrinsic muscles that we can see throughout the pad (Fig. 4). To enable the extra force to move the whiskers forward and overcome drag underwater, the extrinsic muscles, Pars interna profunda, and Pars maxillaris probably also play an important role (Fig. 5). The movement of the whiskers forward will be important for positioning them towards salient stimuli in the environment (Arkley et al. 2014; Grant and Arkley 2016) such as toward hydrodynamic stimuli for foraging (Adachi et al. 2022) and complex environmental stimuli to guide locomotion (Arkley et al. 2014).

Compared to terrestrial mammals such as the Gray Short-tailed Opossum (*Monodelphis domestica*; Grant et al. 2013a) or Norway Rat (*Rattus norvegicus*; Haidarliu et al. 2010; Haidarliu et al. 2024), superficial layers of the Pars interna profunda and Pars maxillaris form clear lines between the whisker rows and run the entire length of the mystacial pad in *L. lutra*, especially in the more ventral whisker rows (Figs 4a and 5d). A similar extrinsic muscle arrangement has only been described before in the phocid *P. vitulina* (Elder et al. 2025a). However, these were formed of the muscles *M. nasolabialis* and *M. maxillolabialis*, which sit deep within the mystical pad; they similarly formed lines between the follicles but attached caudally. We did not observe any caudally attached extrinsic muscles here (Fig. 5). However, as in *P. vitulina*, protraction of the superficial layers of Pars interna profunda and the Pars maxillaris between the follicles will likely translate the whole mystacial pad and move the whiskers forward, causing a bunching or rounding of the pad at the same time (Elder et al. 2025a). This process can clearly be seen in *L. lutra* by comparing retraction video stills (Fig. 6c) to protraction video stills (Fig. 6a and b) where the yellow silhouette of the face is visibly curved during protraction—and can also be seen in the contact-induced asymmetry example (Fig. 6d) where the protracting side of the face contralateral to the initial whisker contact is more curved than the ipsilateral side, which is also less protracted. Therefore, these muscles are likely to play a role in both bilateral and unilateral protractions. Extrinsic muscles are thought to be more variable between species (Yohro 1977; Grant et al. 2013b). It would certainly be interesting to see if lines of extrinsic muscles between follicles are common in the mystacial pads of other aquatic species, especially to uncover which species have deep caudally attached muscles and which, like the otter, have rostrally attached and more superficial fibers. The otariids would be of particular interest to study, as no species of the group has had their whisker muscles described yet.

Contact-induced asymmetry is a whisker control behavior that enables light touches on the side ipsilateral to the initial whisker contact and increases the number of whisker contacts on the side contralateral to the initial contact (Mitchinson et al. 2007). The otter that we studied could control both sides of their whiskers independently, successfully performing this behavior. However, asymmetry was usually caused only by the reduction of ipsilateral whisker protraction and rarely by an increase in contralateral protraction (Table 2). Contact-induced asymmetry is a common behavior across whiskered mammals and has been observed in most species that have been studied so far, apart from European Hedgehog (*Erinaceus europaeus*), Red Fox (*Vulpes vulpes*), Domestic Rabbit (*Oryctolagus cuniculus domesticus*), and Domestic Guinea Pig (*Cavia porcellus*; Grant et al. 2018; Grant et al. 2023). *Lutra lutra*, studied here, did not engage in spread reduction, which reduces the spread between whiskers and is thought to increase the number of whisker contacts with an object (Grant et al. 2009). However, this behavior and the associated extrinsic muscles have only been observed in rodents so far and are unlikely to occur in species outside of rodents (Grant et al. 2009, 2013b, 2017; Haidarliu et al. 2024). Nevertheless, observing both whisker movements and asymmetry of movements, coupled with the adaptations in whisker extrinsic musculature, reveals the importance of whisker movement and control for *L. lutra*. This is especially evident when observing *L. lutra* during locomotion and foraging, when their whiskers can clearly be seen protracting and moving (Fig. 1, right-hand panels).

### Whisker movements

The whisker movements measured in the otter in the present study were largely comparable to those reported from pinnipeds in similar tactile textural discrimination tasks. The whisker angular positions of *L. lutra* (84° to 87°) were lower than those of South African fur seals (*Arctocephalus pusillus*, 108° to 110°; Nakhwa et al. 2024) and *P. vitulina* (103° to 113°; Milne et al. 2020; Nakhwa et al. 2024), but higher than California sea lions (*Zalophus californianus*; 58° to 61°; (Milne et al. 2020; Milne et al. 2021; Table 4). In terms of whisker amplitude, the amplitude range in *L. lutra* of 20° to 28° was lower than *A. pusillus* (38° to 41°; Nakhwa et al. 2024) and *P. vitulina* (27° to 34°; Milne et al. 2020; Nakhwa et al. 2024), but comparable to Z. *californianus* (19° to 36°; Milne et al. 2020; 2021; Table 4). The whisker asymmetry of *L. lutra* (8° to 22°) overlapped with that of *P. vitulina* (7° to 9°) and *A. pusillus* (16° to 17°; Nakhwa et al. 2024), suggesting similar levels of asymmetric whisker movements during active tactile discrimination (Table 4). Indeed, observing the whisker movements of *L. lutra* suggests that they are in no way smaller or less controlled than those of pinnipeds, providing evidence for the importance of studying the whiskers of Lutrinae.

**Table 4:** Comparative metric of whisker movement parameters: angular position, amplitude asymmetry, and decision times of the Eurasian Otter (*Lutra lutra*; current study), Sea Otter (*Enhydra lutris*), and pinniped species from other studies during similar tactile textural discrimination tasks.

Species	Angular position (°)	Whisker amplitude (°)	Asymmetry (°)	Decision time (s)	Behavioral context	Reference
**Eurasian Otter (*Lutra lutra*)**	84° to 87°	20° to 28°	8° to 22°	0.01 to 0.13	Texture discrimination task	Current study
**Sea Otter (*Enhydra lutris*)**	Not reported	Not reported	Not reported	∼0.10 to 0.30	Paws vs vibrissae tactile textural discrimination	Strobel et al. (2018)
**South African Fur seal (*Arctocephalus pusillus*)**	108° to 110°	38° to 41°	16° to 17°	Not reported	Texture discrimination task	Nakhwa et al. (2024)
**Harbor Seal (*Phoca vitulina*)**	103° to 113°	27° to 34°	7° to 9°	<0.40 (size discrimination)	Texture discrimination task	Grant et al. 2013a; Milne et al. (2020); Nakhwa et al. (2024)
**California Sea Lion (*Zalophus californianus*)**	58° to 61°	19° to 36°	Not reported	∼0.27 to 0.33	Texture discrimination tasks	Milne et al. (2020, 2021)

The *L. lutra* studied here could learn a texture discrimination task in about 12 sessions. He was able to make choices for textures quickly, with all decisions occurring in less than 0.2 s (means of 0.098 to 0.132 s), which was similar to those reported in *E. lutris* (∼0.1 to 0.3 s; Strobel et al. 2018), while being faster than those observed in *P. vitulina* and *Z. californianus* (∼0.3 to 0.5 s and ∼0.27 to 0.33 s, respectively; Grant et al. 2013a; Milne et al. 2021), and nearly 25 times faster than human fingertips (<∼5 s; Strobel et al. 2018) during similar texture discrimination tasks (Table 4). Decision times of *L. lutra* were slower on the textured surfaces compared to the smooth surface, suggesting that identifying the smooth distractor texture by tactile touch was easier than discriminating between the 2 different textured surfaces (Table 3).

It must be noted that the texture task developed here is stationary and artificial, compared to dynamic whisker-use in the wild. Therefore, our values of whisker movements and control may not be truly representative of whisker-use in the wild. However, they provide a useful comparison to other species that have been studied using similar experimental designs. Developing comparative studies in the field has not yet been done, although it would be a useful step in understanding whisker-use, especially during natural foraging, social, and locomotion behaviors (Grant 2025, 2026).

### Whisker shape and layout

Whisker base radius, length, and taper of *L. lutra* contributed to explaining most of the variance related to the first PC of whisker shape (50%; Table 1). Whisker base radius and taper were both similar to values that have been previously described for pinnipeds (see Fig. 3 in Dougill et al. 2023), and larger than those of terrestrial mammals. This pattern suggests that the whiskers of *L. lutra* are thicker and more tapered than those of terrestrial mammals, which is likely to make them stiffer underwater (Dougill et al. 2023). Indeed, recently, we found that the material of the whisker (the modulus of elasticity) is stiffer in grey seals (*Halichoerus grypus*) and *L. lutra* compared to a terrestrial *V. vulpes* (Grant et al. 2025), suggesting how important whisker stiffness is for aquatic species, probably to ensure precise positioning underwater. However, despite these adaptations in shape, we observe that the whisker and medulla cross-sections are circular in *L. lutra*, and we see no cross-sectional adaptations such as being oval or having undulations that can be found in the pinnipeds (Ginter et al. 2012; Milne et al. 2022).

As with all mammalian whiskers (Grant and Arkley 2016), *L. lutra* whiskers were arranged into a grid, including 7 whisker rows and 8 whisker columns, including a straddler row (Fig. 3). The more rostral whiskers were smaller and more densely packed than the caudal whiskers, especially the microvibrissae (Fig. 3). The rostral whiskers usually contacted stimuli during the texture discrimination task (Fig. 6), which has also been observed in *P. vitulina* during a size discrimination task (Grant et al. 2013a) and likely provides a higher-resolution sampling area by contacting these more densely packed whiskers, serving to increase the numbers of whiskers contacted.

The ventral 3 rows were notably more organized than the dorsal 5 rows, both in terms of follicle and muscle arrangement, suggesting that there might be a compartmentalization of the whisker pad. Compartmentalization of mystacial whisker pads was first described by Yamakado and Yohro (1979) in mice, who identified a nasal compartment (rows A and B) and a maxillary compartment (ventral rows) of whiskers that develop from different growth centers in an embryo. Different growth centers are also likely to be responsible for the nasal and maxillary divisions in the whisker pad of *L. lutra* and might potentially be a common feature across mammals since it has also been observed in marsupials (Grant et al. 2013b). We observed much more variation in the nasal (dorsal) compartment of the pad compared to the ventral compartment (Fig. 3b, c). It might be that there are individual differences in whisker layout in *L. lutra*, especially in the nasal compartment, as has been previously described in lions (Pennycuick and Rudnai 1970), polar bears (Anderson et al. 2007), and sea lions (Osterrieder et al. 2017). This possibility may mean that whisker positions of the nasal compartment could be used for individual identification of *L. lutra.* However, it is challenging to identify the mystacial whiskers from nasal and microvibrissae (Fig. 3), and the thick fur makes it hard to spot small or newly growing whiskers, which may account for some of the observed individual differences here (Fig. 3c). Nevertheless, it would be interesting to further investigate the uniqueness of whisker arrangements in otters and other mammalian species, especially where individual identification can be challenging; for example, in the absence of coat markings such as spots and stripes.

Not many other species of Lutrinae have had their whiskers studied, apart from *N. vision*, which was also found to have whiskers with relatively large base radii that were circular in cross-section (Dougill et al. 2020), and *E. lutris*, whose whisker shape were not specifically described but also appears to be circular in cross-section (Marshall et al. 2014). We propose that semi-aquatic Lutrinae species are likely to have stiffer whiskers than terrestrial species to ensure precise positioning underwater, and we recommend that this could be tested in future studies. Species of otters, including *E. lutris* and Asian Short-Clawed Otter (*Aonyx cinereus*), are relatively common in zoo collections worldwide, and perhaps should be another focus of future whisker behavior work.

Only the pinnipeds have, thus far, been found to have whiskers with oval cross-sections, and only the phocids have undulating whiskers. These undulations are thought to suppress acoustic noise and wind sounds (Liu et al. 2022; Chen et al. 2025), vortex-induced vibrations (Hanke et al. 2010), and help to shed heat away from the vibrissae (Yoon et al. 2020; Grant 2026). Therefore, undulating phocid seal whiskers may be more robust to aspects of environmental change, including acoustic pollution, changing flow regimes, and increasing temperatures, compared to the whiskers of other species. Smooth and circular whiskers, such as those of the otter and all other mammals, are likely to be less robust to these changes. Changing land-use patterns and habitats can impact sediment distribution and nutrient cycling in the aquatic environment, increasing the turbidity of the water and reducing reliance on vision. Whisker sensing could easily help compensate somewhat for vision in semiaquatic mammals such as otters. Understanding more about whisker form and function, therefore, has important implications for the conservation of charismatic, keystone mammalian whisker specialists such as pinnipeds and otters.

## Supplementary Material

gyag021_Supplementary_Data

## Supplementary data

**Supplementary Data SD1**. Table of mean±SD. whisker shape variables for each of the individual otters.
