# Supplementary material for: Describing the whiskers of a semi-aquatic caniform: the Eurasian Otter (Lutra lutra)
Source: J Mammal. 2026 Apr 9;107(3):450–61. doi: 10.1093/jmammal/gyag021 (PMC13271763; doi:10.1093/jmammal/gyag021)
Supplement: gyag021_Supplementary_Data [file gyag021_supplementary_data.docx]

Supplementary Data S1. Table of mean±s.d. whisker shape variables for each of the individual otters.

|  | **Otter 1** | **Otter 2** | **Otter 3** | **Otter 4** | **Otter 5** |
| --- | --- | --- | --- | --- | --- |
| **Curvature A** | 0.43±0.90 | 0.16±1.65 | 0.40±1.11 | 0.09±0.54 | 0.24±0.89 |
| **Curvature B** | 0.16±0.64 | 0.29±0.51 | 0.18±0.56 | 0.19±0.36 | 0.29±0.51 |
| **Base radius** | 0.0078±0.0017 | 0.0115±0.0042 | 0.0103±0.0033 | 0.0010±0.0036 | 0.0085±0.0029 |
| **Taper ω1** | -0.0053±0.0021 | -0.0077±0.0033 | -0.0073±0.0024 | 0.0064±0.0030 | 0.0058±0.0027 |
| **Length** | 33.62±14.97 | 24.54±16.44 | 16.09±7.43 | 21.80±11.88 | 23.86±13.33 |
| **PC1** | 0.95±1.21 | -0.51±2.13 | -0.43±1.18 | -1.42±1.45 | 0.25±1.41 |
| **PC2** | -0.22±1.26 | 0.23±1.39 | 0.35±1.44 | -0.01±0.87 | -0.29±1.27 |
